# Effects of a Novel Blood Glucose Forecasting Feature on Glycemic Management and Logging in Adults With Type 2 Diabetes Using One Drop: Retrospective Cohort Study

**DOI:** 10.2196/34624

**Published:** 2022-05-03

**Authors:** Steven D Imrisek, Matthew Lee, Dan Goldner, Harpreet Nagra, Lindsey M Lavaysse, Jamillah Hoy-Rosas, Jeff Dachis, Lindsay E Sears

**Affiliations:** 1 One Drop New York, NY United States

**Keywords:** blood glucose forecast, health forecasting, machine learning, model, precision health, blood glucose, blood glucose logging, type 2 diabetes, forecast, mHealth, digital health, smartphone, precision, monitoring, cohort, retrospective, diabetes

## Abstract

**Background:**

Personalized feedback is an effective behavior change technique frequently incorporated into mobile health (mHealth) apps. Innovations in data science create opportunities for leveraging the wealth of user data accumulated by mHealth apps to generate personalized health forecasts. One Drop’s digital program is one of the first to implement blood glucose forecasts for people with type 2 diabetes. The impact of these forecasts on behavior and glycemic management has not been evaluated to date.

**Objective:**

This study sought to evaluate the impact of exposure to blood glucose forecasts on blood glucose logging behavior, average blood glucose, and percentage of glucose points in range.

**Methods:**

This retrospective cohort study examined people with type 2 diabetes who first began using One Drop to record their blood glucose between 2019 and 2021. Cohorts included those who received blood glucose forecasts and those who did not receive forecasts. The cohorts were compared to evaluate the effect of exposure to blood glucose forecasts on logging activity, average glucose, and percentage of glucose readings in range, after controlling for potential confounding factors. Data were analyzed using analysis of covariance (ANCOVA) and regression analyses.

**Results:**

Data from a total of 1411 One Drop users with type 2 diabetes and elevated baseline glucose were analyzed. Participants (60.6% male, 795/1311; mean age 50.2 years, SD 11.8) had diabetes for 7.1 years on average (SD 7.9). After controlling for potential confounding factors, blood glucose forecasts were associated with more frequent blood glucose logging (*P*=.004), lower average blood glucose (*P*<.001), and a higher percentage of readings in range (*P*=.03) after 12 weeks. Blood glucose logging partially mediated the relationship between exposure to forecasts and average glucose.

**Conclusions:**

Individuals who received blood glucose forecasts had significantly lower average glucose, with a greater amount of glucose measurements in a healthy range after 12 weeks compared to those who did not receive forecasts. Glucose logging was identified as a partial mediator of the relationship between forecast exposure and week-12 average glucose, highlighting a potential mechanism through which glucose forecasts exert their effect. When administered as a part of a comprehensive mHealth program, blood glucose forecasts may significantly improve glycemic management among people living with type 2 diabetes.

## Introduction

Diabetes currently affects an estimated 10.5% of Americans, while recent projections indicate its prevalence is increasing worldwide [1,2]. While complications from diabetes range from microvascular-related organ and peripheral tissue damage to death [1], the majority of people with diabetes do not adequately manage their blood glucose [3]. In recent years, mHealth apps have attempted to promote self-care behaviors that are critical for the management of type 2 diabetes (T2D) with mixed success. Compared to non–app users, mHealth app users with diabetes report higher levels of self-care behaviors [4]. A meta-analysis pooling results from randomized controlled trials (RCTs) of 9 mHealth apps found all 9 apps effective in improving diabetes-related outcomes, reducing hemoglobin A_1c_ (HbA_1c_) by a mean 0.49% [5]. Meanwhile, several other reviews note studies with weaker or no effects for diabetes-related outcomes [6-10]. The fact that mHealth apps have realized such varied success raises the question of which components or features are most effective in driving outcomes.

While mHealth apps for diabetes have a range of different features [11], modules for logging diabetes-related data such as blood glucose are among the most common, with many apps also enabling the logging of food, physical activity, and medications [12]. Logging as a form of self-monitoring, delivered along with feedback, constitutes a behavior change technique, which is a systematic procedure included as an active component of an intervention designed to change behavior. Among mHealth apps, it has been noted that self-regulation techniques, such as self-monitoring, goal setting, and performance feedback, are the most frequently utilized [13,14]. Given the theoretical impact of such behavior change techniques on health behavior and clinical outcomes, mHealth apps have the opportunity to incorporate this logged health information and deliver personalized feedback to their users [15,16]. In a previous study, incorporating live feedback from a diabetes coach in response to hypoglycemic or hyperglycemic events showed success [17]. Further, a meta-analysis comparing apps with a feedback component versus those without this feature found that only apps delivering feedback were effective in reducing HbA_1c_ [7]. To our knowledge, mHealth apps with self-monitoring and a feedback component have exclusively focused on past behavior and outcomes. As mHealth apps scale and accumulate a larger repository of data, methods of providing immediate, specific, and personalized feedback about the future are a worthwhile avenue to explore. Data science techniques, such as artificial intelligence, machine learning, and predictive analytics, have been simultaneously described as the next frontier in mHealth apps and also as one of the greatest challenges facing them; these innovations may be the drivers of a personalized and automated feedback mechanism [18].

There are few existing examples of machine learning used to forecast future events for persons with diabetes. In one example, the need for pharmacological therapy was forecast for patients with gestational diabetes [19]. In another study, infections and hypoglycemic events were accurately forecast for individuals with type 1 diabetes (T1D) [20,21]. Although they indicated that diabetes outcomes could be effectively forecast, these studies focused solely on the development and validation of predictive models. The application of predictive models within a diabetes intervention has not previously been tested, to our knowledge. In 2018, One Drop (Informed Data Systems Inc) validated a machine learning model for blood glucose forecasts and subsequently provided the tool to users with T2D in the One Drop app. When forecasts are delivered, they may be paired with behavioral suggestions, such as going for a walk and retesting blood glucose. Multiple studies have established the effectiveness of One Drop for people with diabetes; program participation has been associated with reductions in self-reported, estimated, and lab-tested HbA_1c_, average blood glucose, self-reported hyperglycemic symptoms, diabetes distress, and self-efficacy, [22-24] with preliminary RCT data showing effects on lab-tested HbA_1c_, diet, activity, and depression among persons diagnosed with T2D and hypertension. One Drop’s blood glucose forecasts have demonstrated high accuracy and acceptability, with approximately 92% of users finding them helpful [25,26]. Three years after its inception, One Drop remains the sole mHealth app delivering blood glucose forecasts to its users. The effectiveness of these forecasts has yet to be established.

The current retrospective cohort study evaluated the impact of One Drop’s 1- to 8-hour blood glucose forecasts on logging behavior and clinical outcomes among individuals with T2D and elevated average blood glucose who used the One Drop app over a 12-week period. First, we evaluated the effects of exposure to glucose forecasts on average blood glucose and percentage points in range (%PIR) by comparing participants who did or did not receive the forecasts. Second, we tested a potential behavioral mechanism through which the blood glucose forecasts exert their effect by examining their impact on glucose logging behavior. Lastly, we investigated a potential mediated relationship in which forecasts were associated with glucose management through the mechanism of glucose logging.

## Methods

### One Drop Intervention

One Drop is a multi-condition mHealth solution that can be tailored to each user’s unique needs and preferences. The One Drop digital platform targets people with prediabetes, T1D or T2D, high blood pressure, high cholesterol, or combinations of these conditions. The One Drop mobile app can be used standalone or in conjunction with a monthly or yearly blood glucose test strip subscription, Bluetooth-enabled One Drop blood glucose meter, or connected devices (eg, Wi-Fi–enabled weight scales or Wi-Fi–enabled smart blood pressure monitors).

The One Drop mobile app is available for devices running the iOS, Android, or WatchOS operating systems. Uses can opt to enroll in the free or premium versions of the app. The free version includes extensive logging functionality with the capacity to log health data (eg, blood glucose, blood pressure, HbA_1c_, and weight), intensity and duration of exercise, food eaten, and medications prescribed and taken. When users begin logging data actively (ie, manually through the app or with a synced One Drop meter) or passively (via integrations with Apple HealthKit or Google Fit), detailed reports and visualizations become available, displaying summaries of the entered data and blood glucose trends over time. Messages of support, health-related insights, and educational content are also delivered to users’ in-app inboxes or newsfeeds. The premium One Drop app subscription additionally provides users with on-demand access to health coaching with certified health professionals specializing in their conditions, machine learning–powered trends and forecasts, adjustable goal setting, and a personalized content library with hundreds of lessons.

### Blood Glucose Forecasts

**Figure 1:**
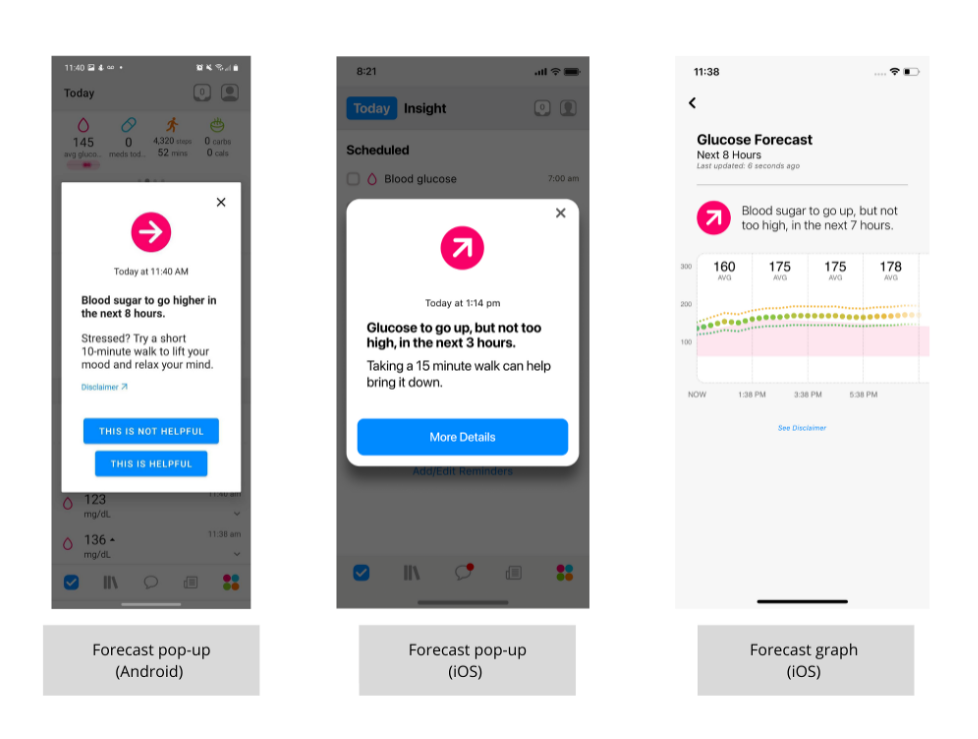
Sample blood glucose forecast in the One Drop app.

Blood glucose forecasts were introduced to the One Drop app as a free feature for users with T2D in September 2018 and became a premium feature in August 2020. These forecasts project the direction (ie, up, steady, or down) that a user’s blood glucose will trend in the following 1 to 8 hours. Users automatically begin receiving blood glucose forecasts upon recording their first blood glucose reading and continue to receive them after each subsequent recording. When a forecast is generated, users receive a pop-up notification indicating the direction (rising, steady, or falling blood glucose) and duration (1-8 hours) of the forecast. These notifications can be paired with an actionable suggestion to help maintain healthy blood glucose levels. Figure 1 shows an example of a blood glucose forecast in the One Drop app.

Users who joined One Drop prior to September 2018 or did not have a paid subscription after August 2020 did not have access to the blood glucose forecasts. After forecasts were implemented in the program, users could become ineligible to receive forecasts if they failed to meet any of the prediction algorithm’s requirements. Users did not receive forecasts if (1) they had extremely high glucose (>600 mg/dL); (2) they had extremely high glucose variability (>80 mg/dL forecast standard error); (3) their logging frequency was consistent with continuous glucose monitor (CGM) use (>50 readings in a 24-hour period); (4) they had ever recorded bolus insulin use; (5) they recorded basal insulin use prior to March 2019 (ie, the time when the model began serving predictions to these users).

### Study Design and Procedures

This study employed a retrospective cohort design, evaluating real-world outcomes over a 12-week period; participants were thus not recruited for study participation. There were 2 cohorts that were compared after statistically controlling for available potential confounders. The first cohort received at least one blood glucose forecast in weeks 1 through 11 and the second received no forecasts. Those included in the group who received no forecasts did not have a paid subscription after August 2020 or were ineligible to receive blood glucose forecasts based on the exclusion criteria.

On August 10, 2021, One Drop users with T2D who had one or more blood glucose measurements recorded 12 weeks following their first recorded reading were identified. Blood glucose readings were entered either manually through the app or passively through the One Drop blood glucose meter, another synced device, or integration with Apple Health Kit or Google Fit. The query for eligible users was limited to those who had started using One Drop and recorded their blood glucose for the first time in 2019 or later to minimize the possibility of comparing groups that participated in different iterations of One Drop. Additionally, only users with at-risk baseline blood glucose (estimated HbA_1c_≥7%) were queried. Users with a glucose logging frequency consistent with CGM use were excluded. The baseline measurement time point was defined by averaging the first 7 days of blood glucose readings, beginning with the first recorded blood glucose reading for that individual. Follow up (in week 12) consisted of measurements recorded from days 77 to 83.

### Study Oversight

One Drop received an exemption for institutional review board approval and a waiver of informed consent from Solutions IRB, an independent ethics review company (Little Rock, AR and Yarnell, AZ) to study all deidentified data owned by One Drop. User data are stored in a secure cloud-based server. All One Drop users must actively agree to an end user license agreement upon creation of their accounts, granting One Drop permission to use data entered in the app for analysis, reporting, and research purposes.

### Measurements

#### Group

The total number of forecasts received between weeks 1 and 11 was summed in order to group users according to whether they had received any blood glucose forecasts during the study period. Those with zero forecasts in that time period were placed in the “did not receive forecasts” group (n=177). Those with ≥1 forecast were placed in the “received forecasts” group (n=1234).

#### User Characteristics

Date of birth, gender, diabetes type, insulin use, and year of diagnosis are self-reported in the One Drop app, though not all users provide these data. Age was calculated as the number of months between a user’s date of birth and the date of their first recorded blood glucose measurement divided by 12. Users taking insulin were identified based on whether they had recorded taking a dose of basal or bolus insulin on or before the date of their first blood glucose log. The number of years diagnosed with diabetes was calculated as the difference between the user-reported year of diagnosis and the year of a user’s first recorded blood glucose measurement.

#### Logging Activity

Logging activity was measured as the number of blood glucose entries recorded in each user’s first week, as well as the number of entries in the 11-week period prior to the follow-up week.

#### Blood Glucose Variability Measurement

Glycemic variability is commonly calculated as the standard deviation of an individual’s glucose values over time [26,27]. The standard deviation of a user’s blood glucose recordings during the first week was calculated to express individual baseline blood glucose variability. Users were required to have at least 3 readings recorded in week 1 to have a blood glucose variability metric computed.

#### Average Glucose Measurement

One Drop’s database consists of real-world data; both biologically impossible (eg, 0 mg/dL) and implausible (eg, above 600 mg/dL, which is beyond the measuring capacity of a glucometer) readings can be entered. The range of plausible measurements was defined as 30 mg/dL to 600 mg/dL. Readings outside of this range were not included in average glucose calculations. For baseline average glucose, an average of all plausible blood glucose measurements in week 1 was calculated. Similarly, for follow-up average glucose, all plausible blood glucose recordings in a user’s week 12 were averaged. In order to identify the at-risk population in terms of A_1c_, average glucose values were translated to an estimated percentage of glycated hemoglobin (eHbA_1c_) using the following formula: eHbA_1c_ = (average glucose + 46.7) / 28.7 [28]. The American Diabetes Association recommends a goal of <7% HbA_1c_ for most people with diabetes; achieving this level is associated with a reduced risk for diabetes-related complications [29]. Users with an eHbA_1c_ of ≥7%, corresponding to ≥154.2 mg/dL average glucose, were classified as at risk.

To visualize potential interactions, a multi-categorical variable was created to further classify those with eHbA_1c_≥7% into four categories, indicating an increasing risk of complications from elevated blood glucose concentration: (1) 8%> eHbA_1c_ ≥7%; (2) 9%> eHbA_1c_ ≥8%; (3) 10%> eHbA_1c_ ≥9%; and (4) eHbA_1c_ ≥10%.

#### %PIR Measurement

Research has demonstrated that blood glucose levels falling below 70 mg/dL or rising above 180 mg/dL are associated with increased risk for diabetes-related complications [30]. Percentage of blood glucose points in range (%PIR) is a metric adapted from the CGM-specific metric time in range, applied to measurements obtained from manual blood glucose meters. A 10% change in %PIR has been associated with a change in HbA_1c_ of 0.4% [31].

Blood glucose values were considered in range if they fell between 70-180 mg/DL. Each user’s %PIR was calculated by dividing the number of blood glucose measurements in range by the total number of recorded blood glucose measurements, for both week 1 and week 12.

### Cohort Selection

There were 1411 users included in the analysis. Users were deemed eligible for analysis if (1) they reported a diagnosis of T2D; (2) their first week of One Drop participation was between the years 2019 to 2021; (3) they recorded ≥3 blood glucose measurements in their first week; (4) they recorded ≥1 blood glucose measurement in week 12; (5) they had a self-reported year of diagnosis; and (6) their calculated week-1 HbA_1c_ was 7.0%.

Users averaging more than 7 blood glucose measurements per day for the first 11 weeks of the study period were assumed to be using a CGM (>539 measurements; 7 measurements times 7 days times 11 weeks) and were excluded.

### Analyses

All analyses were performed using SPSS Version 28 (IBM Corp). Our predetermined α level was .05 for all statistical tests. Between-group differences in the year users started One Drop, age, gender, years diagnosed with T2D, insulin use, number of week-1 blood glucose recordings, sum of week-1 to week-11 blood glucose recordings, baseline average glucose, and baseline individual blood glucose variability were assessed. Differences were tested with 2-tailed independent-samples *t* tests for continuous variables and chi-square tests for categorical variables. Descriptive statistics were also computed for these variables. A gender of “other” was reported by 4 users in the group who did not receive forecasts and zero users in the group who received forecasts; this value was treated as missing and excluded from the chi-square analysis assessing between-group differences in gender.

Variables were omitted from models if high missingness was observed (greater than 50%), but descriptive statistics for the variable are still reported. Age was the only variable meeting this threshold (1143/1411, 81% missing).

We specified 2 analysis of covariance (ANCOVA) models to determine the forecast group effect on blood glucose outcomes. Covariates were selected for ANCOVAs if their baseline values were significantly different in the groups who received and did not receive forecasts. The first ANCOVA tested the group effect on week-12 average glucose, controlling for years diagnosed, insulin use, baseline blood glucose variability, and baseline average glucose. The second ANCOVA tested the group effect on week-12 %PIR, controlling for years diagnosed, insulin use, baseline blood glucose variability, and baseline %PIR. Interactions between the group variable and covariates were tested; significant interactions were held and interpreted, while nonsignificant interactions were dropped from the final reported models.

**Figure 2:**
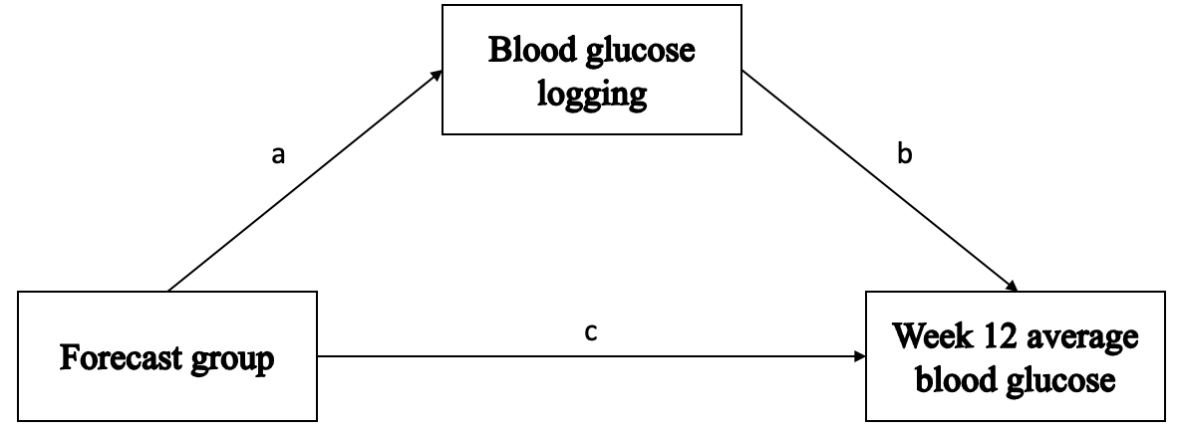
Hypothesized model of how blood glucose logging mediates the effect of group on week-12 average glucose.

The secondary analysis sought to find mechanisms through which exposure to blood glucose forecasts resulted in greater reductions in average glucose concentration in week 12; it was hypothesized that blood glucose logging could be one such mechanism. This hypothesized model is illustrated in Figure 2. To test this hypothesis, 3 separate linear regression models were specified to establish that the direct effect of group on blood glucose logging behavior and the direct effect of group and logging behavior on week-12 average glucose were all significant.

PROCESS is a free software add-on for SPSS that includes over 70 predefined models [32]. Mediation models in PROCESS incorporate ordinary least squares regression and estimate indirect effects and their confidence intervals through a bootstrapping procedure that is robust against nonnormal sample distributions [33]. Model 4 was specified to estimate the indirect effect of group on week-12 average glucose via blood glucose logging with 5000 bootstrap samples. All models included years diagnosed, insulin use, baseline blood glucose variability, and baseline average glucose as covariates.

## Results

A total of 1411 users were included in the analyses. The users were 60.6% male (795/1311), aged 12 to 84 years old (mean age 50.2 years, SD 11.8), diagnosed with T2D for <1 to 46 years (mean 7.1 years, SD 7.9), and recorded between 3 and 78 blood glucose logs (mean 14.5, SD 9.2) in their first week and between 3 and 532 (mean 122.0, SD 86.1) logs in the 11 weeks before follow up.

The groups were significantly different in several baseline variables. Compared to users who received forecasts, those who did not receive forecasts had a higher average likelihood of reporting insulin use (14.1% versus 6.2%; *χ*^2^_1_=14.8; *P*<.001), more years diagnosed with T2D (10.4 years, SD 9.1 versus 6.6 years, SD 7.6; *t*_1409_=6.00; *P*<.001), higher baseline average glucose (256.16 mg/dL, SD 82.36 versus 206.23 mg/dL, SD 48.37; *t*_1409_=11.55; *P*<.001), higher baseline blood glucose variability (58.64 mg/dL, SD 29.63 versus 43.91 mg/dL, SD 23.42, *t*_1409_=7.55; *P*<.001) and a lower baseline percentage of blood glucose logs in range (26.96%, SD 28.91% versus 41.98%, SD 29.9%; *t*_1409_=–6.27; *P*<.001). The groups did not differ in the year they began using One Drop (*P*=.21), age (*P*=.07), gender (*P*=.24), or number of week-1 blood glucose logs (*P*=.08). Descriptive statistics and *P* values from tests of baseline differences are presented in Table 1.

**Table 1:** Sample characteristics with tests of difference by group.

Characteristics		Total (N=1411)	Received forecasts (n=1234)	Did not receive forecasts (n=177)	*P* value^a^	
**Year started One Drop, n (%)**						.21
	2019	681 (48.3)	597 (48.4)	84 (47.5)		
	2020	614 (43.5)	530 (42.9)	84 (47.5)		
	2021	116 (8.2)	107 (8.7)	9 (5.1)		
**Gender, n (%)^b^**						.24
	Male	795 (60.6)	701 (61.2)	94 (56.6)		
	Female	512 (39.1)	440 (38.4)	72 (43.4)		
	Other	4 (0.3)	4 (0.3)	0 (0)		
Insulin use, n (%)		101 (7.2)	76 (6.2)	25 (14.1)	<.001	
Age (years), mean (SD)		50.2 (11.8)	49.8 (11.4)	54.5 (14.7)	.07	
Years diagnosed with T2D^c^, mean (SD)		7.1 (7.9)	6.6 (7.6)	10.4 (9.1)	<.001	
**Blood glucose logs, mean (SD)**						
	Week-1 blood glucose logs	14.5 (9.2)	14.7 (9.4)	13.4 (7.9)	.08	
	Week-1 to week-11 blood glucose logs	122.0 (86.1)	124.1 (87.0)	107.2 (79.0)	.01	
**Glycemic management, mean (SD)**						
	Week-1 average blood glucose (mg/dL)	212.50 (56.27)	206.23 (48.37)	256.16 (82.36)	<.001	
	Week-1 blood glucose variability (mg/dL)	45.76 (24.76)	43.91 (23.42)	58.64 (29.63)	<.001	
	Week-1 points in range (%)	40.10 (30.18)	41.98 (29.90)	26.96 (28.91)	<.001	

^a^From chi-square test or 2-tailed independent-samples *t* test.

^b^“Other” was treated as a missing value and excluded from the chi-square analysis.

^c^T2D: type 2 diabetes

### Primary Outcome 1a: Average Glucose

The ANCOVA revealed significant mean differences between the groups in week-12 average glucose when controlling for baseline average glucose and covariates (*F*_7_=40.75, *P*<.001). All model covariates except insulin use (*P*=.16) were significant (years diagnosed, baseline average glucose, and baseline blood glucose variability; all *P*<.001). Additionally, significant interaction effects for group × baseline average glucose (*F*_1_=5.28, *P*=.02) and group × baseline blood glucose variability (*F*_1_=15.12, *P*<.001) were observed.

**Figure 3:**
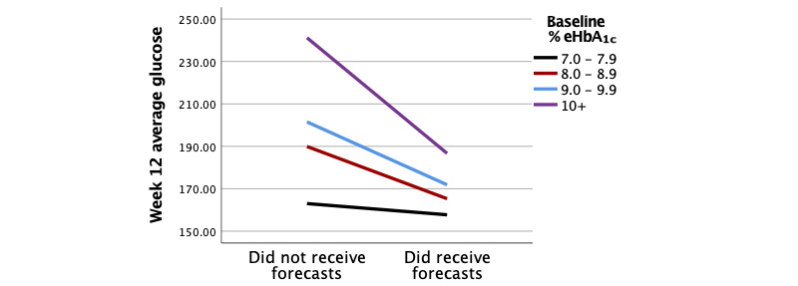
Interaction diagram of the effects of group and baseline average glucose on week-12 average glucose.

To explore the group × baseline average glucose interaction, ANCOVA models were specified to evaluate the group effect at different levels of baseline eHbA_1c_. Baseline average glucose was split into eHbA_1c_ risk levels for interpretability. This interaction is visualized in Figure 3. Users receiving forecasts ended week 12 with significantly lower average glucose than those not receiving forecasts when baseline eHbA_1c_ was ≥10% (mean difference –54.52mg/dL; –1.9% eHbA_1c_, *P*=.002). Nonsignificant results within the other three categories may be attributed to low power (1-*β*<.4). Among those with ≥8% eHbA_1c_, reductions in week-12 eHbA_1c_ ranged from –0.86% to –1.9%.

**Figure 4:**
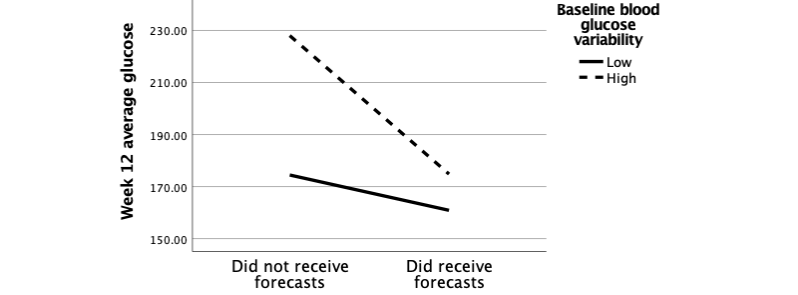
Interaction diagram of the effects of group and baseline glucose variability on week-12 average glucose.

The second interaction, group × baseline glucose variability, was evaluated by performing a median split on baseline blood glucose variability and specifying ANCOVA models for each level. Values below the median were categorized as “low variability” and values above the median were categorized as “high variability.” Values at the median were pruned. Among users with high baseline blood glucose variability, those receiving forecasts experienced significant reductions in week-12 average glucose (mean difference –53.22mg/dL; –1.85% eHbA_1c_, *P*<.001) relative to those not receiving forecasts. Figure 4 visualizes the group × baseline glucose variability interaction.

### Primary Outcome 1b: %PIR

The ANCOVA revealed a significant mean difference between groups in week-12 %PIR when controlling for covariates (*F*_6_=40.27, *P*<.001). All model covariates except insulin use (*P*=.3) were significant (years diagnosed, baseline %PIR, and baseline blood glucose variability; all *P*<.001). Additionally, a significant interaction effect between group × baseline %PIR (*F*_1_=4.84, *P*=.03) was observed.

**Figure 5:**
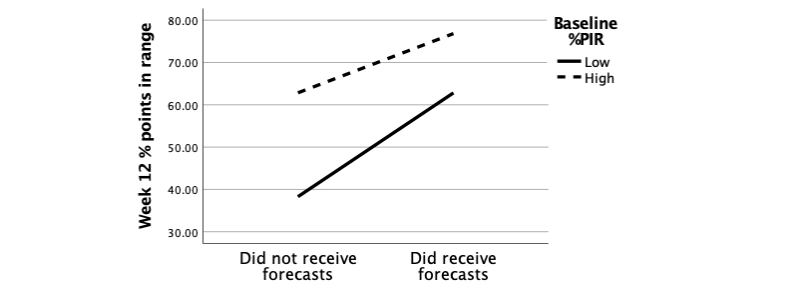
Interaction diagram of the effects of group and baseline average percentage points in range on week-12 average glucose.

The group × baseline %PIR interaction was evaluated by performing a median split on baseline %PIR and specifying ANCOVA models for each level. Values below the median were categorized as “low %PIR” and values above the median were categorized as “high %PIR.” Values at the median were pruned. Among users with low baseline %PIR, those receiving forecasts experienced a significant increase in %PIR (mean difference .45%, *P*<.001) compared to those not receiving forecasts. A visualization of the group × baseline %PIR interaction is presented in Figure 5.

### Secondary Analysis: Mediation of Group Effect on Average Glucose by Blood Glucose Logging Behavior

As reported above, the results suggest that users receiving blood glucose forecasts experienced greater reductions in week-12 average glucose than those not receiving forecasts. We thus proceeded with our secondary analysis.

The forecast group was significantly and positively associated with total blood glucose logs (path a; *b*=20.72, *t*_1405_=2.86, *P*=.004). Total blood glucose logs were significantly associated with week-12 average glucose (path b; *b*=–0.13, *t*_1405_=–6.86, *P*<.001). The total effect of group on week-12 average glucose was also significant (path c; *b*=–21.74, *t*_1405_=–4.27, *P*<.001). As all 3 paths were significant, we proceeded by regressing week-12 average glucose on group, controlling for total blood glucose logs. The direct effect was reduced, but remained significant (*b*=–19.22, *t*_1404_=–3.82, *P*<.001).

**Figure 6:**
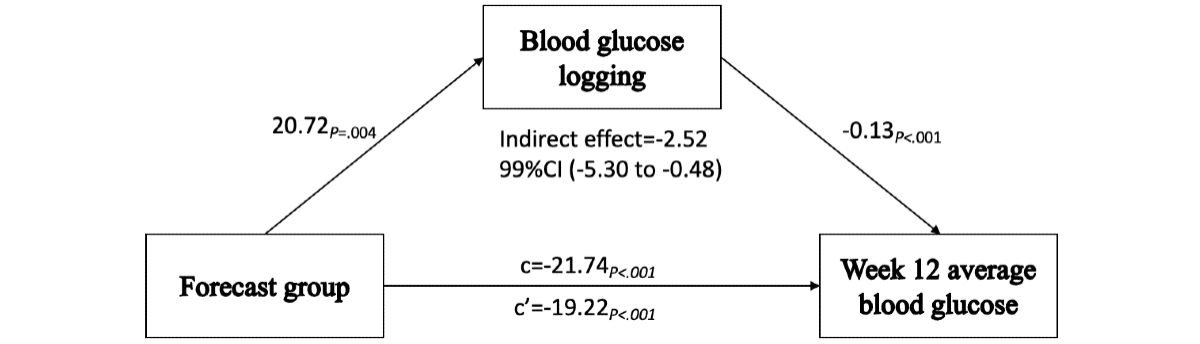
Mediation analysis. Path values are unstandardized regression coefficients. The indirect effect was calculated using 5000 bootstrap samples with a 99% CI.

Results from the bootstrapping procedure produced an estimated indirect effect (path c’) of group on week 12 average glucose through total blood glucose logs with a 99% CI that did not include 0, indicating a significant mediation effect. While the indirect effect was significant, the direct effect also remained significant, indicating that blood glucose logging is a partial mediator of the relationship. Mediation results are summarized in Table 2. The conceptual model is presented in Figure 6, along with unstandardized path coefficients.

**Table 2:** Results of mediation analysis.

Mediation analysis^a^		*b* (SE)	*P* value
**Model**			
	Path from group to blood glucose logging	20.72 (7.24)	.004
	Path from group to week-12 average glucose	–21.74 (5.09)	<.001
	Path from blood glucose logging to week-12 average glucose	–0.13 (0.02)	<.001
**Effect**			
	Direct effect of group on week-12 average glucose	–19.22 (5.03)	<.001
	Indirect effect of group on week-12 average glucose	–2.52 (0.91; 99% CI –5.30 to –0.48)	

^a^Model summary: *R^2^*=0.18; *P*<.001.

## Discussion

In this retrospective cohort study, One Drop users with T2D who received blood glucose forecasts had significantly lower average glucose after 12 weeks than those who did not receive the forecasts, after accounting for group differences at baseline. This effect was most pronounced for users with high baseline blood glucose or high baseline blood glucose variability. Additionally, among users who had low baseline %PIR, those who received blood glucose forecasts had significantly higher %PIR after 12 weeks than those who did not receive blood glucose forecasts. Over the course of the study period, participants who received forecasts logged their glucose significantly more frequently than those who did not receive forecasts. Our secondary analysis suggests that the forecasts encouraged users to log their blood glucose more often, which in turn was associated with lower blood glucose at week 12.

These results have potential implications for the health care costs of individuals with diabetes. The average yearly cost of medical care for persons with diabetes is US $9600 [34]. A systematic review found that mHealth interventions for T2D were cost-effective [35]. When adjusted for inflation, a one-point reduction in HbA_1c_ is associated with a US $1376.51 reduction in patient costs [36]. In this study, among users with ≥8% eHbA_1c_, the reduction in week-12 average glucose would translate to an estimated patient cost savings of US $1183.80 to $2615.37 per year. Among those with high baseline blood glucose variability, glucose reductions would translate to an estimated patient cost savings of US $2546.54 per year. Among those with low baseline %PIR, exposure to forecasts was associated with a 45% increase in %PIR. This increase in %PIR is associated with an eHbA_1c_ reduction of 1.8%, representing a potential US $2477.72 cost savings [31,36]. These cost savings are incremental increases for those receiving forecasts compared to those not receiving forecasts. Previous research highlighting One Drop’s association with reductions in lab-tested HbA_1c_ was conducted prior to the advent of blood glucose forecasts; therefore, actual cost savings for those participating in this study’s iteration of One Drop may be even higher.

### Strengths and Limitations

The data generated for this study were collected from users of an mHealth app, and thus have real-world generalizability; however, the rapid evolution of the product, the available data, and a lack of experimental controls are limitations. First, there may have been covariates to consider that were not available for analysis, such as age, race, ethnicity, socioeconomic status, health motivation, CGM use, and other factors that may have differed across the groups and impacted outcomes. The impact of these variables on the relationship between blood glucose forecasts and diabetes outcomes is a potential avenue for future research.

Additionally, the groups systematically differed at baseline due to prespecified criteria that excluded some users from receiving forecasts. Those who received forecasts had fewer years diagnosed with T2D, lower glucose, higher %PIR, lower glucose variability, and were less likely to be taking insulin than those who did not receive forecasts. While we controlled for these variables, the bias inherent to this study design may still have been present. The results should be interpreted with this potential for selection bias in mind.

Finally, because the One Drop users included in this study participated at different times over a three-year period, it is possible that they participated in different iterations of the One Drop app that differentially impacted their app experiences, creating potential confounders. The One Drop app is continually updated and improved based on clinical science, behavior science, and research performed internally and externally. Further, participation in this study was limited to 12 weeks. While other studies of mHealth interventions have evaluated glucose outcomes at 3 months [37,38], insight into the sustained impact of forecasts on glucose beyond a 12-week period is limited. To address these limitations, which are characteristic of real-world evidence, future long-term, prospective randomized studies are needed to confirm the causal impact of forecasts on self-monitoring behavior and glucose management.

While the study design and the nature of real-world data likely introduced bias in the results, real-world studies confer unique benefits that extend beyond the confines of a controlled study. Studies using real-world data exchange the internal validity of an RCT for external validity [39], allowing our results to be generalized to other populations with T2D using mHealth apps. The stringent requirements for inclusion in RCTs may exclude participants that would normally be seen in a real-world clinical setting [40]. When used in conjunction with evidence-based clinical practice, real-world evidence has shown that mHealth apps can lead to significant improvements in glycemic management over the course of 1 year [41]. Aside from supporting an existing care network, mHealth apps may also have a niche in providing care for hard-to-reach populations [42].

### Blood Glucose Forecasts

A major innovation in data-powered health insights is the predictive modeling of specific outcomes, such as blood glucose levels. Although predictive models do not necessarily reveal causes and effects, these models have been used for discovery, hypothesis testing, risk prediction, and the identification of counterfactuals and effective interventions [43]. Despite promising evidence on machine learning models, such as one study that demonstrated a significantly reduced glycemic response when a machine learning model was paired with a dietary intervention, blood glucose forecasts remain a nascent technique. There are currently no agreed-upon protocols for machine learning models in precision health [44,45], and a number of different models have been used as frameworks for machine learning training and development [46]. Further, there is no well-defined approach to estimate carbohydrate intake, the effect of stress and activity on blood glucose level, or the portability of machine learning models to capture inter- and intraindividual variability [45,47].

Until now, the majority of blood glucose forecast research has been focused on T1D [48]. This has limited the scope and real-world potential for blood glucose forecasts, as most (90.9%) diabetes cases in the United States have been diagnosed as T2D [49]. Blood glucose forecast research for T2D has mainly focused on forecasting the future incidence of disease or adverse glycemic events [50]. In addition, blood glucose forecast studies for either T1D or T2D have largely been limited to a short forecast horizon of 2 hours or less [48]. The current study advances the literature in studying a forecast horizon of 8 hours in an at-risk T2D population.

### Conclusion

One Drop is one of the first mHealth apps to provide blood glucose forecasts to its users; our findings are the first to provide evidence for the effectiveness of delivering blood glucose forecasts as part of an mHealth intervention. The results suggest that exposure to blood glucose forecasts may be effective for individuals with T2D who have a high level of blood glucose, with forecast exposure associated with reduced glucose, a higher percentage of blood glucose %PIR, and increased self-monitoring of blood glucose. Further, blood glucose logging was a partial mediator of the relationship between forecast exposure and glucose reduction, highlighting a potential mechanism through which forecast exposure is associated with reduced glucose. Taken together, this novel evidence highlights the potential for One Drop blood glucose forecasts to improve glycemic management in individuals with T2D.

## Abbreviations

%PIR: percent of blood glucose points in range
ANCOVA: analysis of covariance
CGM: continuous glucose monitor
eHbA_1c_: estimated percentage of glycated hemoglobin
HbA_1c_: glycated hemoglobin
mHealth: mobile health
RCT: randomized controlled trial
T1D: type 1 diabetes
T2D: type 2 diabetes

## References

[1] (2020). National diabetes statistics report. Centers for Disease Control and Prevention.

[2] Khan MAB, Hashim MJ, King JK, Govender RD, Mustafa H, Al Kaabi J (2020). Epidemiology of Type 2 Diabetes - Global Burden of Disease and Forecasted Trends. J Epidemiol Glob Health.

[3] American Diabetes Association (2021). 5. Facilitating Behavior Change and Well-being to Improve Health Outcomes. Diabetes Care.

[4] Kebede MM, Pischke CR (2019). Popular Diabetes Apps and the Impact of Diabetes App Use on Self-Care Behaviour: A Survey Among the Digital Community of Persons With Diabetes on Social Media. Front Endocrinol (Lausanne).

[5] Hou C, Carter B, Hewitt J, Francisa T, Mayor S (2016). Do Mobile Phone Applications Improve Glycemic Control (HbA1c) in the Self-management of Diabetes? A Systematic Review, Meta-analysis, and GRADE of 14 Randomized Trials. Diabetes Care.

[6] Maharaj A, Lim D, Murphy R, Serlachius A (2021). Comparing Two Commercially Available Diabetes Apps to Explore Challenges in User Engagement: Randomized Controlled Feasibility Study. JMIR Form Res.

[7] Cui M, Wu X, Mao J, Wang X, Nie M (2016). T2DM Self-Management via Smartphone Applications: A Systematic Review and Meta-Analysis. PLoS One.

[8] Doupis J, Festas G, Tsilivigos C, Efthymiou V, Kokkinos A (2020). Smartphone-Based Technology in Diabetes Management. Diabetes Ther.

[9] Frazetta D, Willet K, Fairchild R (2012). A systematic review of smartphone application use for type 2 diabetic patients. OJNI.

[10] Eberle C, Löhnert M, Stichling S (2021). Effectiveness of Disease-Specific mHealth Apps in Patients With Diabetes Mellitus: Scoping Review. JMIR Mhealth Uhealth.

[11] Angelini S, Alicastro G, Dionisi S, Di Muzio M (2019). Structure and Characteristics of Diabetes Self-management Applications: A Systematic Review of the Literature. Comput Inform Nurs.

[12] Jimenez G, Lum E, Car J (2019). Examining Diabetes Management Apps Recommended From a Google Search: Content Analysis. JMIR Mhealth Uhealth.

[13] Direito A, Dale LP, Shields E, Dobson R, Whittaker R, Maddison R (2014). Do physical activity and dietary smartphone applications incorporate evidence-based behaviour change techniques?. BMC Public Health.

[14] Middelweerd A, Mollee JS, van der Wal CN, Brug J, Te Velde SJ (2014). Apps to promote physical activity among adults: a review and content analysis. Int J Behav Nutr Phys Act.

[15] Park H, Cormier E, Gordon G, Baeg J (2016). Identifying Health Consumers' eHealth Literacy to Decrease Disparities in Accessing eHealth Information. Comput Inform Nurs.

[16] van Deursen AJAM, van Dijk JAGM (2011). Internet skills performance tests: are people ready for eHealth?. J Med Internet Res.

[17] Downing J, Bollyky J, Schneider J (2017). Use of a Connected Glucose Meter and Certified Diabetes Educator Coaching to Decrease the Likelihood of Abnormal Blood Glucose Excursions: The Livongo for Diabetes Program. J Med Internet Res.

[18] Istepanian RS, Al-Anzi T (2018). m-Health 2.0: New perspectives on mobile health, machine learning and big data analytics. Methods.

[19] Velardo C, Clifton D, Hamblin S, Khan R, Tarassenko L, Mackillop L (2021). Toward a Multivariate Prediction Model of Pharmacological Treatment for Women With Gestational Diabetes Mellitus: Algorithm Development and Validation. J Med Internet Res.

[20] Woldaregay AZ, Launonen IK, Årsand E, Albers D, Holubová A, Hartvigsen G (2020). Toward Detecting Infection Incidence in People With Type 1 Diabetes Using Self-Recorded Data (Part 1): A Novel Framework for a Personalized Digital Infectious Disease Detection System. J Med Internet Res.

[21] Daskalaki E, Nørgaard K, Züger T, Prountzou A, Diem P, Mougiakakou S (2013). An early warning system for hypoglycemic/hyperglycemic events based on fusion of adaptive prediction models. J Diabetes Sci Technol.

[22] Osborn CY, van Ginkel JR, Marrero DG, Rodbard D, Huddleston B, Dachis J (2017). One Drop | Mobile on iPhone and Apple Watch: An Evaluation of HbA1c Improvement Associated With Tracking Self-Care. JMIR Mhealth Uhealth.

[23] Kumar S, Moseson H, Uppal J, Juusola JL (2018). A Diabetes Mobile App With In-App Coaching From a Certified Diabetes Educator Reduces A1C for Individuals With Type 2 Diabetes. Diabetes Educ.

[24] Osborn CY, van Ginkel JR, Rodbard D, Heyman M, Marrero DG, Huddleston B, Dachis J (2017). One Drop | Mobile: An Evaluation of Hemoglobin A1c Improvement Linked to App Engagement. JMIR Diabetes.

[25] Goldner D, Heyman M, Hirsch A (2019). 49-LB: Reported Utility of Automated Blood Glucose Forecasts.

[26] Suh S, Kim JH (2015). Glycemic Variability: How Do We Measure It and Why Is It Important?. Diabetes Metab J.

[27] Frontoni S, Di Bartolo P, Avogaro A, Bosi E, Paolisso G, Ceriello A (2013). Glucose variability: An emerging target for the treatment of diabetes mellitus. Diabetes Res Clin Pract.

[28] Nathan D, Kuenen J, Borg R, Zheng H, Schoenfeld D, Heine R (2008). Translating the A1C assay into estimated average glucose values. Diabetes Care.

[29] American Diabetes Association (2021). 9. Pharmacologic Approaches to Glycemic Treatment: Standards of Medical Care in Diabetes—2021. Diabetes Care.

[30] Beck R, Bergenstal R, Riddlesworth T, Kollman C, Li Z, Brown AS, Close KL (2019). Validation of Time in Range as an Outcome Measure for Diabetes Clinical Trials. Diabetes Care.

[31] Cutruzzolà A, Irace C, Parise M, Fiorentino R, Pio Tripodi PF, Ungaro S, Babinsky V, Gnasso A (2020). Time spent in target range assessed by self-monitoring blood glucose associates with glycated hemoglobin in insulin treated patients with diabetes. Nutr Metab Cardiovasc Dis.

[32] Hayes A (2017). Introduction to mediation, moderation, and conditional process analysis: A regression-based approach.

[33] Preacher KJ, Hayes AF (2008). Asymptotic and resampling strategies for assessing and comparing indirect effects in multiple mediator models. Behav Res Methods.

[34] American Diabetes Association (2018). Economic Costs of Diabetes in the U.S. in 2017. Diabetes Care.

[35] Rinaldi G, Hijazi A, Haghparast-Bidgoli H (2020). Cost and cost-effectiveness of mHealth interventions for the prevention and control of type 2 diabetes mellitus: A systematic review. Diabetes Res Clin Pract.

[36] Grabner M, Abbott S, Nguyen M, Chen Y, Quimbo R (2013). Estimated Cost Savings Associated With A1c Reductions In A Large Us Commercial Health Plan. Value Health.

[37] Agarwal P, Mukerji G, Desveaux L, Ivers NM, Bhattacharyya O, Hensel JM, Shaw J, Bouck Z, Jamieson T, Onabajo N, Cooper M, Marani H, Jeffs L, Bhatia RS (2019). Mobile App for Improved Self-Management of Type 2 Diabetes: Multicenter Pragmatic Randomized Controlled Trial. JMIR Mhealth Uhealth.

[38] Zimmermann G, Venkatesan A, Rawlings K, Scahill MD (2021). Improved Glycemic Control With a Digital Health Intervention in Adults With Type 2 Diabetes: Retrospective Study. JMIR Diabetes.

[39] Luce B, Drummond M, Jönsson B, Neumann PJ, Schwartz JS, Siebert Uwe, Sullivan SD (2010). EBM, HTA, and CER: clearing the confusion. Milbank Q.

[40] Blonde L, Khunti K, Harris SB, Meizinger C, Skolnik NS (2018). Interpretation and Impact of Real-World Clinical Data for the Practicing Clinician. Adv Ther.

[41] Tu Y, Chang Y, Chiou H, Lai K (2021). The Effects of Continuous Usage of a Diabetes Management App on Glycemic Control in Real-world Clinical Practice: Retrospective Analysis. J Med Internet Res.

[42] Steinman L, van Pelt M, Hen H, Chhorvann C, Lan CS, Te V, LoGerfo J, Fitzpatrick AL (2020). Can mHealth and eHealth improve management of diabetes and hypertension in a hard-to-reach population? -lessons learned from a process evaluation of digital health to support a peer educator model in Cambodia using the RE-AIM framework. mHealth.

[43] Pearson TA, Califf RM, Roper R, Engelgau MM, Khoury MJ, Alcantara C, Blakely C, Boyce CA, Brown M, Croxton TL, Fenton K, Green Parker MC, Hamilton A, Helmchen L, Hsu LL, Kent DM, Kind A, Kravitz J, Papanicolaou GJ, Prosperi M, Quinn M, Price LN, Shireman PK, Smith SM, Szczesniak R, Goff DC, Mensah GA (2020). Precision Health Analytics With Predictive Analytics and Implementation Research: JACC State-of-the-Art Review. J Am Coll Cardiol.

[44] Zarkogianni K, Litsa E, Mitsis K, Wu P, Kaddi CD, Cheng C, Wang MD, Nikita KS (2015). A Review of Emerging Technologies for the Management of Diabetes Mellitus. IEEE Trans Biomed Eng.

[45] Prosperi M, Min JS, Bian J, Modave F (2018). Big data hurdles in precision medicine and precision public health. BMC Med Inform Decis Mak.

[46] Kavakiotis I, Tsave O, Salifoglou A, Maglaveras N, Vlahavas I, Chouvarda I (2017). Machine Learning and Data Mining Methods in Diabetes Research. Comput Struct Biotechnol J.

[47] Zeevi D, Korem T, Zmora N, Israeli D, Rothschild D, Weinberger A, Ben-Yacov O, Lador D, Avnit-Sagi T, Lotan-Pompan M, Suez J, Mahdi JA, Matot E, Malka G, Kosower N, Rein M, Zilberman-Schapira G, Dohnalová L, Pevsner-Fischer M, Bikovsky R, Halpern Z, Elinav E, Segal E (2015). Personalized Nutrition by Prediction of Glycemic Responses. Cell.

[48] Contreras I, Vehi J (2018). Artificial Intelligence for Diabetes Management and Decision Support: Literature Review. J Med Internet Res.

[49] Bullard KM, Cowie CC, Lessem SE, Saydah SH, Menke A, Geiss LS, Orchard TJ, Rolka DB, Imperatore G (2018). Prevalence of Diagnosed Diabetes in Adults by Diabetes Type - United States, 2016. MMWR Morb Mortal Wkly Rep.

[50] Saunajoki AE, Auvinen JP, Bloigu AH, Timonen MJ, Keinänen-Kiukaanniemi SM (2020). Evaluating the 1-h post-load glucose level to predict future type 2 diabetes. Diabetes Res Clin Pract.

